# Ventilation of St. James's Hall, Piccadilly

**Published:** 1858-07

**Authors:** 


### VENTILATION OF ST. JAMES'S HALL, PICCADILLY.

We confine our notice of this building chiefly to the ventilation of the great hall. The body of the room is 94 feet long and 60 feet wide; and its height to the crown of the ceiling, which is semi-cylindriform, is 59 feet 3 inches. At one end, in addition, is the orchestra recess, 43 feet 6 inches in diameter, enclosed by a semi-circular wall; and at the opposite end another recess containing two galleries. The extreme length is 139 feet. The room is arranged to seat conveniently an audience of 1700 and an orchestra of 400 persons. It has a capacity of 338 thousand cubic feet, which is equal to 161 cubic feet of air to each person.

The ventilation is effected by perforated panels in the ceiling. In the large ceiling above the body of the room are thirty-two such panels, arranged in four rows of eight panels each, lengthwise of the hall. Above these perforations zinc tubes are carried up to above the slated roof, and covered with zinc tops. It is these tubes and caps which give the singular appearance to the building, as seen from Regent Street and Piccadilly. In the crown of the ceiling of the gallery recess is one more perforated panel.

In consequence of the curved form of the ceiling, the two side rows of perforated panels are at a level of 6 feet 3 inches lower than the central rows, and on a level with that in the ceiling of the gallery recess. The effect of this arrangement of apertures is that the cold air of the atmosphere descends through the perforations at the lower level, and the heated air ascends through those at the upper level. The fresh air is warmed and diffused in its descent, while its velocity is expended. To give full effect to this action, ingress of air at the doors is prevented as far as possible. When it is desirable, as during hot weather, to maintain a temperature inside as even as possible with the outside air, the doors are kept open, whereby all the apertures in the ceilings become outlets of air. By this arrangement, the room has been very nearly as cool as the outside.

The hall is lighted by sixty-eight gaseliers, diffused at equal spaces over the body of the room, and suspended 21 feet below the ceiling; each gaselier has eight burners radiating horizontally. The only provision especially for warming is two open fireplaces in the side walls.

